# Doing Justice: Ethical Considerations Identifying and Researching Transgender and Gender Diverse People in Insurance Claims Data

**DOI:** 10.1007/s10916-024-02111-w

**Published:** 2024-10-12

**Authors:** Ash B. Alpert, Gray Babbs, Rebecca Sanaeikia, Jacqueline Ellison, Landon Hughes, Jonathan Herington, Robin Dembroff

**Affiliations:** 1 Yale Cancer Center, 333 Cedar Street, WWW 205, New Haven, CT 06511 USA; 2 Yale School of Medicine, New Haven, CT USA; 3 Department of Public Health Sciences, University of Rochester Medical Center, Rochester, NY USA; 4 Department of Health Services, Policy, and Practice, Brown University School of Public Health, Providence, RI USA; 5 Department of Philosophy, University of Rochester, Rochester, NY USA; 6 Department of Health Policy and Management, School of Public Health, University of Pittsburgh, Pittsburgh, PA USA; 7 Center for Innovative Research On Gender Health Equity, Department of General Internal Medicine, University of Pittsburgh School of Medicine, Pittsburgh, PA USA; 8 Department of Population Medicine, Harvard Pilgrim Health Care Institute, Boston, MA USA; 9 Department of Epidemiology, Harvard T.H. Chan School of Public Health, Boston, MA USA; 10 Department of Health Humanities and Bioethics, University of Rochester Medical Center, Rochester, NY USA; 11 Department of Philosophy, Yale University, New Haven, CT USA

**Keywords:** Transgender Persons, Epistemic Injustice, Ethics, Insurance Claim Review, Administrative Data

## Abstract

Data on the health of transgender and gender diverse (TGD) people are scarce. Researchers are increasingly turning to insurance claims data to investigate disease burden among TGD people. Since claims do not include gender self-identification or modality (*i.e.,* TGD or not), researchers have developed algorithms to attempt to identify TGD individuals using diagnosis, procedure, and prescription codes, sometimes also inferring sex assigned at birth and gender. Claims-based algorithms introduce epistemological and ethical complexities that have yet to be addressed in data informatics, epidemiology, or health services research. We discuss the implications of claims-based algorithms to identify and categorize TGD populations, including perpetuating cisnormative biases and dismissing TGD individuals’ self-identification. Using the framework of epistemic injustice, we outline ethical considerations when undertaking claims-based TGD health research and provide suggestions to minimize harms and maximize benefits to TGD individuals and communities.

## Background

Transgender and gender diverse (TGD) health data are scarce [1]. While this scarcity is often attributed to insufficient gender identity data collection, a more fundamental cause is structural oppression of TGD people. TGD health research has been undermined by insufficient funding, barriers for researchers who are TGD, and dangers—including state-sponsored harassment [2] and physical harm [3]—for TGD people who disclose their identities. Due to these barriers, most studies of TGD people have been based on convenience samples and are cross-sectional or retrospective [1]. Studies that use convenience samples may only capture TGD people receiving care at lesbian, gay, bisexual, transgender, and queer-focused clinics [4]. Cross-sectional survey-based studies are limited because researchers cannot reliably estimate the overall United States TGD population and thus cannot appropriately calculate survey weights [4]. Researching TGD health using insurance claims provides one mechanism to address the limitations of other data. [5–9]

Insurance claims capture population-level data on covered beneficiaries and the clinical services they have received. They provide large sample sizes without the burden of primary data collection [10]. Claims data often have several limitations including lack of diagnostic (*e.g.,* disease stage) and physiological (*e.g.,* hemoglobin A1C values) detail, missing data related to churn, and poor variable quality for measures not related to reimbursement [10]. Researching TGD people with claims presents unique limitations including the lack of self-reported gender identity or modality (*i.e.,* TGD or not) [11] in claims. To address this limitation, researchers use algorithms based on diagnosis (*e.g.,* gender identity disorder), procedure (*e.g.,* phalloplasty), and prescription codes (*e.g.*, estradiol) to identify TGD people [5–9] and methods to stratify subjects by inferred sex assigned at birth (SAAB) and gender self-identification [8, 9]. For example, researches might infer that an individual who had a hysterectomy and is on testosterone has the sex assigned at birth “female” or the gender identity “trans masculine or nonbinary.” [8] Little scholarship considers the epistemological assumptions or ethical implications of claims-based TGD health research. As multiethnic TGD researchers, scholars, and allies based in the Eastern and mid-Western United States, many of who study TGD health with claims and other methods, we ae invested in ensuring that our own and others’ research aligns with ethical principles. We thus describe the epistemic dangers of TGD claim-based research and the algorithms necessary for such research and call for improvements to its ethics [12].

## Epistemic Injustice Toward Transgender People

Medicine is steeped in cisnormativity [13]. Cisnormative assumptions in medicine include the assumption that SAAB is the most medically-relevant aspect of a person and that their gender presentation, identification, and behavior will align with social expectations based on their SAAB [14–16]. One example is the assumption that someone with the SAAB “female” will have long hair, identify as a “woman,” have ovaries, and want to retain breast tissue. Such assumptions position cisgender people as “normal,” erase or pathologize TGD people, and contribute to epistemic injustices that harm TGD people and undermine the accuracy of research [17, 18].

Epistemic injustice in medicine includes testimonial injustice, or a failure to believe the words of marginalized people including TGD patients [17, 19]. For instance*,* clinicians may assume patients’ symptoms are due to gender-affirming interventions despite patients’ testimony to the contrary [20, 21]. Hermeneutical injustice occurs when TGD people and clinicians lack language to articulate TGD experiences [17]. For example, TGD people who do not see themselves as men or women may be unfamiliar with the term “nonbinary” and thus may struggle to explain their gender to researchers or clinicians. Both testimonial and hermeneutical injustice may also contribute to testimonial smothering, whereby TGD people distort or hide their experiences or identities to access healthcare or avoid healthcare because of risks associated with transphobia [22–25].

Because of the impacts of epistemic injustices, data gleaned using insurance claims are limited and distorted and subsequent knowledge generation and interventions risk harming TGD individuals and communities as we describe in further detail in the subsequent sections.

### Limited and Distorted Data

To our knowledge, no insurance claims database includes a contemporaneous, self-reported gender identity or modality [5, 9, 26]. As such, claims-based research predominantly identifies TGD people by diagnosis codes [5–7]. Some researchers take additional steps to confirm TGD status by cross-referencing additional diagnoses (*e.g.,* endocrine disorder not otherwise specified) and gender-affirming surgery procedure codes (*e.g.,* vaginoplasty) or receipt of hormones (*e.g.,* testosterone) discordant with the sex recorded at the time of prescribing (*e.g.,* “female”) [9]. Some researchers further stratify TGD cohorts by inferred SAAB or gender identity using hormone prescriptions, surgeries, and care specific to reproductive anatomy [8, 9]. For example, if a TGD person’s SAAB was determined to be “female,” and they were prescribed a certain dose of testosterone, their gender is assumed to be “transmasculine.” [9].

Insurance claims demonstrate receipt of care that might be considered gender-affirming care. Claims-based TGD epidemiologic research thus often compares disease burden among TGD people who receive gender-affirming care with health outcomes of non-TGD people who do not. [27] Such research may therefore imply that health disparities are related to *medical interventions* rather than *structural stigma,* erasing the impact of social marginalization [28] and falsely suggesting that gender-affirming care worsens rather than improves outcomes, for example that testosterone increases the risk of metabolic syndrome [29]. Claims-based TGD algorithms also undercount people not known to be TGD by their clinicians and TGD people not using prescribed gender-affirming care, including those with intersectional stigma and resultant barriers to care, which may lead to the erroneous conclusions that TGD people are more likely to be White and living in the Northeast or West [7, 30]. Because individuals with more healthcare interactions are more likely to be categorized as TGD, claims-based algorithms may also oversample those with diagnosed illnesses and artificially inflate TGD morbidity and mortality, for example suggesting that TGD people have higher overall morbidity and higher rates of every medical condition [6, 31].

Claims-based algorithms that categorize TGD subjects by gender or SAAB risk epistemic harms for TGD people as well as data inaccuracies. Researchers have developed such algorithms because administrative claims data do not capture TGD identity and sex markers are inconsistent across data sources and could refer to clinicians’ assumptions, patients’ gender identification, SAAB, or the marker on a legal document (*e.g.*, social security record, insurance card). Researchers use claims-based algorithms to instead infer SAAB based on procedures (*e.g.,* vaginoplasty) and prescriptions (*e.g.,* estradiol) and make further assumptions about gender self-identification.

Medical categorization by SAAB reinforces the concept of SAAB as an immutable fact with biological significance and perpetuates discourse that erroneously uses “sex” as a vague proxy for biological factors such as anatomy, hormones, and karyotype [16]. SAAB is not a reliable proxy for these factors as hormone levels fluctuate, people take exogenous hormones or undergo surgeries, and at least 1% of the population has anatomy, hormonal milieu, or a karyotype that differs from what is assumed based on SAAB [32]. Algorithms grouping people by SAAB overlook biological differences. SAAB also cannot be used to reliably infer gender presentation or social gendered treatment, thus algorithms that classify study participants by inferred gender (*e.g.,* “transfeminine or nonbinary”) [8, 9] misclassify and misportray those with additional genders (*e.g.*, man, two-spirit). These limitations thus distort, overgeneralize, or preempt important epidemiologic conclusions, for example suggesting that higher rates of transgender people are transmasculine [9].

### Reinforcement of Epistemic Injustices

Claims-based algorithms reproduce testimonial injustice against TGD people by undermining TGD people’s agency over their gender categorization (*i.e.,* through presentation, self-description, social relationships). Such algorithms also perpetuate hermeneutical injustice by omitting TGD people’s descriptive language about themselves and their experiences. Insurance claims-based research always has this consequence because, unlike other data sources, data based on insurance claims renders a transgender person’s self-identification irrelevant, focusing instead on diagnoses or medical histories, which do not reflect a transgender person’s testimony.

## Ethical Principles

### Respect for Persons

People who enroll in health insurance do not consent to participation in research and are often unaware of whether and how their data are being used for research purposes. Anonymizing data is typically regarded as sufficient, obviating the need to seek consent from individual participants. The circumstances of claims based TGD research, however, necessitate increased caution for several reasons.

Research should respect the self-determination of the people who contribute data. Claims-based research, which assumes a gender taxonomy limited to “transfeminine and nonbinary” and “transmasculine and nonbinary” [8] erases non-Western and Indigenous gender categories (*e.g.,* Nádleehi)*,*[33, 34] as well as alternative categories developed by TGD people to understand their embodiment and desires (*e.g.,* bigender, butch) [35]. As a result, this research not only risks disrespecting patients’ gender identities (i.e., misgendering), but also reinforces the broader social erasure of these persons [23, 36]. This research often also uses cisgender people as the reference group and is deficit-oriented, perpetuating stigmatizing narratives about TGD communities [6, 31, 37, 38]. Claims-based algorithms may also contribute to pathologizing TGD people by identifying them in terms of medications, surgeries, or diagnoses.

### Beneficence

Research ought to minimize risks of harm and ensure that risks imposed are commensurate with benefits. Claims-based TGD health research may not meet this bar. First, the use of putatively de-identified data involves a small but non-zero risk of reidentification through triangulating multiple data points such as age and geography [39]. To our knowledge, this is a theoretical but plausible risk, particularly as TGD communities are a small portion of the general population. Second, a foundation of claims-based research involves identification of gender-affirming care by people without an explicitly associated diagnosis (*e.g.,* endocrine disorder not otherwise specified) [9]. State-level anti-TGD legislation has escalated dramatically [40] including bills which criminalize gender-affirming care [41] [42] and compel the release of TGD patient records [43]. Claims-based algorithms could facilitate such criminalization of TGD people, their clinicians, and families. In the present political moment, researchers must consider whether the benefits of claims-based research equal or outweigh its potential harms.

### Justice

Justice in research requires researchers to include individuals equitably and allocate the benefits and burdens fairly. Claims-based epidemiological studies document disease prevalence, outcomes, and disparities and may impact resource allocation and set the stage for future interventions [44]. Individual TGD people could benefit from information about disease risks or increased resource allocation. At best, the benefits of claims-based epidemiologic research are non-contemporaneous. While individual TGD people have little to gain and much to lose from epidemiologic research performed with their data and without their knowledge, individual researchers have much to gain. Published research manuscripts are the currency of academia, the quantity of which is used to judge grant applicants and faculty reviewed for promotion and tenure.

Health equity tourism, in other words investigators without relevant skills, knowledge, or positionality responding to “timely and often temporary increases in public interest and resources [45],” is often justified by the urgent need to mitigate harms experienced by marginalized communities, for example “lack of information about [TGD healthcare] represents a critical barrier to improving health and reducing disparities [9] or “by identifying these disparities for [TGD people], we can begin to address them [6]”. Researchers without relevant skills or knowledge or who lack TGD positionality may, however, inadvertently create scholarship that is inaccurate or imposes epistemic injustice. Such work may also create testimonial harm by diluting or drowning out the voices of scholars creating knowledge situated in and informed by TGD communities, eliminating and obscuring the experiences of those scholars and their communities.

## Harm-Reduction Practices

To navigate the ethical challenges of claims based TGD research and reduce harm, we suggest the following practices, many of which can be implemented immediately with little additional labor. First, given that the gold standard, self-reported gender identity and modality data, is not available in claims, rather than describing participants by SAAB or gender, which cannot be known based on claims data, we encourage researchers to explicitly describe the categories utilized (*e.g.,* people with cervixes as evidenced by procedure codes.) This would avoid reinforcing cisnormative assumptions (*i.e.,* by centering SAAB) and increase subjects’ autonomy (*i.e.,* by avoiding naming genders for participants).

Second, we suggest researchers explicitly acknowledge relevant limitations of data as outlined above as well as the dangers of research to TGD communities. Such descriptions would allow researchers to avoid making false or harmful epidemiologic, etiologic, or other conclusions, for example that more TGD people are White or that TGD people are more mentally unwell than non-transgender people.

Third, we urge researchers to use reflexivity by which researchers state their positionality and biases to contextualize their work. Such statements would allow readers to better understand potential biases in research questions or conclusions. Fourth, we suggest that data stewards and researchers take care to prevent identifiability of TGD individuals. Such measures could include data masking or aggregate data reporting.

Fifth, we suggest ensuring that TGD researchers—especially those with multiple marginalized identities—lead or co-lead research, guide analyses, and interrogate the work's ethics and utility. This has the potential to shift power imbalances between researched and researchers and will further ensure that research is conducted with the needs of affected community members at the forefront [46]. This manuscript, for example, would not have been generated without the TGD authors who situated epistemic harms within our own experiences.

Our final suggestion represents a more foundational solution: supplement claims-based research *conducted on* TGD people with community-based participatory research *conducted by and alongside* TGD people, which we believe is best practice for ensuring research is meaningful, actionable, and in the service of TGD people. For example, a coalition of a TGD-serving health center with a local TGD advocacy group performed a needs assessment related to TGD people’s experiences using public accommodations [47]. See Table 1 for a checklist.

**Table 1:** Checklist of Best Practices for Claims-Based Transgender and Gender Diverse Research

o Researchers have made explicit the categories they have utilized to stratify subjects (*e.g.,* with cervixes versus without)
o Researches have removed any references to the sex or gender of subjects unless they have been able to assess these via participants’ self-report
o Researchers have explicitly described the limitations of the data
o Researches have explicitly described potential harms of the data except where so doing could increase the potential for harm
o Researchers have included a reflexivity statement that includes the positionality of the team members and their potential biases
o Researchers taken steps to prevent identifiability of subjects, and these are outlined in the manuscript except where so doing could cause harm
o The research team is led or co-led by transgender and gender diverse people who have guided analyses and interrogated the works’ ethics and accuracy
o The research includes community-based participatory methodology, and this methodology is outlined in the manuscript

The resultant work would be TGD-led and centered, community-engaged research that prioritizes the questions and needs of TGD people, especially those with intersecting marginalized identities. The researchers involved would be transparent about their positionality and biases and the work’s limitations. They would also rigorously protect their subjects’ data privacy. Such research methods may create novel questions. For example, rather than asking how many TGD people have attempted suicide [38], such research may investigate interventions to immediately decrease suicide attempts and completion by TGD people. Alternatively, such research may investigate potential associations between bans against gender-affirming therapy and suicide attempts. Such work would center and prioritize the lived experiences of TGD people, name intersecting sources of oppression, and question status quo power relationships [28]. It would thus be, in methods and content, in the service of epistemic and material justice for TGD people. Such work would also have limitations. For example, it often takes longer to conduct research using community-engaged or participatory methods, thus resulting in fewer manuscripts over the same period. Such methods may not result in questions that require big data or large sample sizes. Academia rewards researchers who publish frequently in high impact journals that often prioritize trials with large sample sizes. Though researches could take several steps to minimize harms of claims-based and other research, prioritizing research that truly benefits TGD communities will require fundamental shifts in academic incentives.

## Data Availability

No datasets were generated or analysed during the current study.
